# What Determines Step-Rate at Work? An Investigation of Factors at the Shift, Worker, Ward, and Nursing Home Levels in Eldercare

**DOI:** 10.1093/annweh/wxab027

**Published:** 2021-06-17

**Authors:** Matthew L Stevens, Kristina Karstad, Svend Erik Mathiassen, Leticia Bergamin Januario, Andreas Holtermann, David M Hallman

**Affiliations:** 1 Department of Musculoskeletal Disorders and Physical Workload, The National Research Centre for the Working Environment, Lersø Parkallé 105, 2100 København Ø, Denmark; 2 Centre for Musculoskeletal Research, Department of Occupational Health Sciences and Psychology, University of Gävle, Kungsbäcksvägen 47, 801 76 Gävle, Sweden; 3 Department of Sports Science and Clinical Biomechanics, University of Southern Denmark, Campusvej 55, 5230 Odense, Denmark

**Keywords:** accelerometry, eldercare workers, mixed-effects regression modelling, physical activity

## Abstract

**Objectives:**

Current knowledge on the determinants of step-rate at different organizational levels is limited. Thus, our aim was to identify, in eldercare, at what workplace level differences in step-rate occur and to identify determinants of workers’ step-rate at these levels.

**Methods:**

Participants were 420 eldercare workers from 17 nursing homes (126 wards) in Denmark. Accelerometry was used to assess step-rate (steps per hour) of workers over multiple shifts. We assessed various determinants at different levels of the workplace, i.e. at the (i) shift, (ii) worker, (iii) ward, and (iv) nursing home levels. Variance components analysis identified the percentage contribution to total variance in step-rate from each respective level. Multi-level linear regression modelling was used to investigate the association between candidate determinants at each level and step-rate.

**Results:**

Differences in eldercare workers’ step-rate occurred primarily between shifts (within workers; 44.9%) and between workers (within wards; 49.1%). A higher step-rate was associated with: (i) weekend and evening shifts (versus weekday/day); (ii) job as a care helper (versus care aide) and an increased proportion of time spent on direct care tasks; (iii) working in a somatic ward (versus dementia), an increased resident–staff ratio and permission to take unscheduled breaks; and (iv) lack of elevators.

**Conclusions:**

We found that nearly all variability in step-rate in eldercare work occurs between shifts (within workers) and between workers (within wards). The main determinants of step-rate were related to the type of shift, type of work tasks, staffing ratio, break policy, and availability of elevators.

**What’s important about this paper**

The aim of this study was to identify, in eldercare, what workplace level differences in step-rate occur and to identify determinants of workers’ step-rate at these levels. We found that nearly all variability in step-rate in eldercare work occurs between shifts (within workers) and between workers (within wards). The main determinants of step-rate were related to the type of shift, type of work tasks, staffing ratio, break policy, and availability of elevators. These organizational factors can be modified to alter step-rate to improve health and wellbeing of eldercare workers.

## Introduction

Eldercare is an important profession, with the European Pillar for Social Rights listing affordable and good quality long-term care services as one its core principles (European Commission, 2017). Furthermore, the demand for these care services is increasing, with the number of Europeans aged 80+ expected to rise from 5% in 2016 to 13% in 2070 (Spasova *et al.*, 2018). One of the challenges in eldercare is the provision of a healthy and capable workforce (Spasova *et al.*, 2018). However, high rates of pain, work-related disability, obesity, and poor cardiovascular fitness among eldercare workers (Luime *et al.*, 2004; Andersen *et al.*, 2012; Jensen *et al.*, 2012; Davis and Kotowski, 2015) are barriers to healthy and sustainable working lives. This limits the ability of our society to fulfil the increasing demand for giving care to the increasing number of elderly (Spasova *et al.*, 2018).

One of the characteristics of eldercare work is a high level of occupational physical activity (OPA) (Karstad *et al.*, 2018). This OPA can be measured in several ways and for a profession that requires a great deal of walking, such as eldercare, a simple and understandable proxy for OPA is the number of steps taken at work or occupational step-rate (Karstad *et al.*, 2018). Outside of occupational studies, a higher step count or step-rate show clear and consistent beneficial relationships with a variety of health conditions (Bassett *et al.*, 2017; Kraus *et al.*, 2019). In contrast, occupational studies show that workers with high occupational step-rates have increased pain, disability and sick leave compared with those walking less (Bot *et al.*, 2007; Hansen *et al.*, 2015; Fimland *et al.*, 2018). This is thought to be explained by the environmental and contextual constraints on steps taken at work in occupations with high OPA, where the amount and type of work to be conducted, how it is organized and/or the culture around the conduct of work limits periods of rest and may provide little flexibility to adapt to symptoms such as pain and fatigue (Korshøj *et al.*, 2015). Thus, balancing the step-rate of workers with sufficient rest is important for worker health, particularly in occupations that have a high step-rate, such as eldercare.

In order to balance the step-rate of eldercare workers we must first understand the determinants of that step-rate. This in turn requires that we understand the organizational determinants of step-rate (Vandelanotte *et al.*, 2015; Prince *et al.*, 2019). Organizational determinants are the environmental, psychosocial, and policy determinants that arise from a particular organizational context (Sallis *et al.*, 2006). Environmental determinants relate to the physical space and objects that the individual works in, such as the building or office layout (Hallman *et al.*, 2018) and the tasks conducted. The psychosocial determinants relate to the psychological and social context in which the individual conducts their work (e.g. the level of support from colleagues), while policy determinants relate to the rules and regulations of the organization (e.g. permission to take breaks) (Sallis *et al.*, 2006).

**Figure 1.**
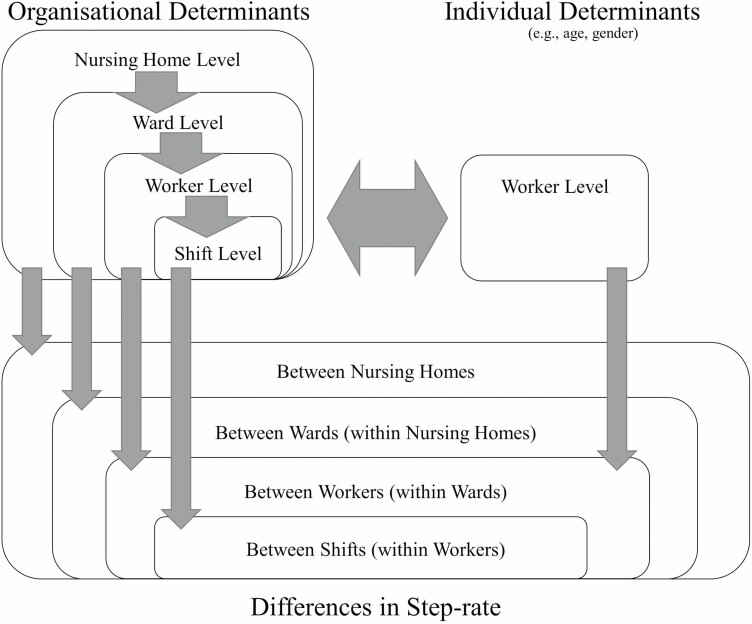
Conceptual diagram of a 4-level hierarchical model on step-rate within eldercare work. This model shows the conceptual split between organizational and individual determinants, the occurrence of organizational determinants across all levels of the 4-level hierarchical model developed, and potential pathways by which the determinants act. Note that the model does not include potential interactions between determinants at different levels, which allow for higher level determinants to affect lower level outcomes.

An important aspect of organizational determinants is that they occur across different organizational levels (Steele and Mummery, 2003; Sallis *et al.*, 2006). In nursing wards, these different levels can be expressed in a 4-level hierarchical model that consists of shifts (within workers), workers (within wards), wards (within nursing homes), and nursing homes (Fig. 1). Accordingly, organizational determinants can be conceptualized as arising from a particular level and are able to affect factors that occur at or below their level. For instance, the presence (or lack of) elevators within a nursing home has the potential to affect the step-rate across all shifts, workers, and wards within that nursing home. Another example is the ward’s resident–staff ratio, which will influence the step-rate of workers within that ward but not outside it (i.e. it will not affect step-rate across the entire nursing home). Note that organizational determinants are not confined to upper organizational levels (e.g. the ward and nursing home levels) but also occur at lower levels. For example, a worker’s job title and the tasks that make up their work are organizational determinants (as they arise from the organizational context) but occur at worker level. Individual determinants (e.g. age and gender) will also occur at the worker level, but although individual determinants are important, they are less useful to understand in the context of making organizational changes to improve worker health. As such our focus throughout this study is on organizational determinants. Once we have a thorough understanding of the organizational determinants of step-rate, we can then use this knowledge in the development of organizational interventions to modify step-rate with the goal of improving the health of eldercare workers.

Current knowledge on the organizational determinants of step-rate is limited (Smith *et al.*, 2016). Moreover, studies that specifically investigate eldercare workers are required to understand the determinants of step-rate specific to eldercare. We have only identified one study (with a total sample of six participants) that addresses organizational determinants of step-rate specifically among eldercare workers (Wakui, 2000). Therefore, the aims of the present study were to: (i) evaluate how much of eldercare workers’ total variance in step-rate occurs at different organizational levels (i.e. between shifts, workers, wards, and nursing homes) and (ii) identify determinants of step-rate at each of these different levels.

## Methods

This study used data from a prospective cohort of eldercare workers collected from September 2013 to December 2014—the Danish Observational Study of Eldercare work and musculoskeletal disorderS (DOSES) (Karstad *et al.*, 2018). Ethical approval for DOSES was provided by the Danish Data Protection Agency and the Ethics Committee for the regional capital of Denmark (H-4-2013-028). As the full details of DOSES have been previously published (Karstad *et al.*, 2018), only aspects specifically relevant to this study will be described below.

### Participants

Eighty-three nursing homes located in Zealand and the capital region of Denmark were purposively selected with the aim of including nursing homes of various sizes and care models, and invited to participate in the study. Twenty nursing homes agreed to participate and were subsequently included. Upon a nursing home agreeing to participate, written information about the aim and activities of the study was distributed to all employees and an information meeting was arranged at the nursing home to inform employees about the study and invite them to participate. Participants in the study were eldercare workers between 18 and 65 years of age, employed in the nursing homes more than 15 h week^−1^ on day and evening shifts, and spent a minimum of 25% of their working time on tasks related to direct care of residents.

### Data collection

Only baseline data were used for this cross-sectional analysis. Baseline data collection for nursing home managers and ward managers consisted of a web-based questionnaire about formal and informal organizational structures at the nursing home and wards. We also collected information about the physical aspects of nursing homes/wards with a ‘workplace walkthrough’, which was conducted together with either a manager or a work and safety representative. Baseline data collection for workers included a structured self-administered questionnaire, a health check (conducted by trained clinical personnel) which recorded technical measures of health and physical capacity, and the collection of work schedules and accelerometer measurements of physical activity at work and leisure.

### Primary outcome

The primary outcome of this study was step-rate (steps per hour) at work, collected using accelerometry. We asked participants to wear three accelerometers (on the thigh, upper back, and dominant arm) for a minimum of four consecutive days including at least two working days. Participants allergic to patches were excluded from wearing the accelerometers. The accelerometers used were ActiGraph GT3X+ accelerometers (ActiGraph, FL, USA). A validated software program (Acti4) (Skotte *et al.*, 2014) was applied for analysing the accelerometer data and counting steps with very high sensitivity and specificity (Ingebrigtsen *et al.*, 2013). Participants were also asked to keep diary recording the time they went to sleep and woke up (time in/out of bed), and when they started and finished work. We then used these diaries to classify the steps recorded into time either in, or outside work and the number of steps occurring in each shift. The step-rate for each shift was then calculated by dividing the number of steps taken by the duration of that specific shift. To be considered a valid representation of a shift, accelerometers needed to collect data for at least 4 h or 75% of that shift. Shifts that did not meet this requirement were excluded from the analysis.

### Determinants at the shift level

All shift-level determinants were derived from the diaries. We classified weekend versus weekday shift from the diaries according to the day most of the time on that shift occurred. The length of each shift (hours) and classification of the type of shift into day, evening, night, or double (day + evening) was also determined using the diaries. Day shifts started between 6 am and 2 pm and ended before 5 pm. Evening shifts started between Midday and 6 pm and ended before 1 am. Night shifts started between 10 pm and 3 am and ended before by 7 am. Double shifts started between 6 am and 9 am and ended between 6 pm and 10 pm.

### Determinants at the worker level

Most worker level determinants were collected in the baseline questionnaire. This included age (years), sex (male/female), country of birth (Denmark/outside Denmark), and the self-reported proportion of time spent conducting: direct care tasks (e.g. feeding, conversing, assisting with practical tasks), support tasks (e.g. cleaning/tidying, kitchen/laundry work), and administration tasks (e.g. meetings, documentation) (5-point Likert scale: rarely/never, roughly 1/4 of the time, roughly 1/2 of the time, roughly 3/4 of the time, almost all the time). We included these tasks as potential determinants, since, while a difference in workers’ step-rate between direct care, support, and administration tasks may appear obvious, it is useful for eventual intervention purposes to understand just how much these tasks do, in fact, differ in terms of step-rate.

Workers’ employment/job was divided into three categories—‘care helpers’ (who had 14 months of training in care provision), ‘care aides’ (who had completed an additional 6 months of training), and ‘nurses or other health professionals’. The extra education undertaken by the care aides (as opposed to care helpers) allows them to independently handle medications (within limits) while helpers do not. In general, care aides also have more responsibility, coordinating different tasks, and information in and between teams, while care helpers are limited to providing care and practical help to the resident.

Information about the psychosocial work environment was collected using questions from the Copenhagen Psychosocial Questionnaire (COPSOQ II) (Pejtersen *et al.*, 2010). The psychosocial dimensions of work collected were: quantitative demands (two items: ‘Do you get behind with your work?’, ‘Do you have enough time for your work tasks?’), influence at work (two items: ‘Do you have a large degree of influence concerning your work?’, ‘Can you influence the amount of work assigned to you?’), social support (two items: ‘How often are your colleagues willing to listen to your problems at work?’, ‘How often do your colleagues talk with you about how well you carry out your work?’), and quality of leadership (four items: ‘To what extent would you say that your immediate superior: -makes sure that the individual member of staff has good development opportunities?’, ‘-gives high priority to job satisfaction?’, ‘-is good at work planning?’, ‘-is good at solving conflicts?’). Each item was collected on a 5-point Likert scale. For the analyses, all items within each dimension were averaged and converted to a 0–100 scale. We collected the information to calculate body mass index (kg m^−2^) at the health check. We assessed step-rate during leisure using accelerometers as per step-rate during work, i.e. averaging all steps at leisure over the total time spent in leisure (excluding sleep time).

### Determinants at the ward and nursing home levels

Determinants at the ward and nursing home level were obtained from ward and nursing home managers using the baseline questionnaire or from the workplace walkthrough. The type of ward was divided into four categories—somatic, dementia, temporary rehabilitation, and independent living. The ward/home size was defined as the maximum number of residents that could be allocated to that ward/home. We obtained information about the usual resident–staff ratio on day and evening shifts by asking ‘what were the usual number of residents on the ward?’ and ‘how many staff are usually working on day [and evening] shifts?’ In the workplace walkthrough we collected information about the availability of rooms for taking breaks (yes/no), whether it was permissible to take breaks from work (yes/no), the number of floors (*n*), and the presence of elevators (yes/no). We also obtained information about the location of various aid devices used by the eldercare workers (1:4 scale; with higher values indicating the device was stored further away from the residents’ rooms). This was done via a combined score that incorporated the responses to questions that asked where these devices were located (in the room, in the corridor, in the ward, outside the ward) for the eight most commonly used aid devices (floor lift, stand-up lift, ceiling lift, sail, easy slide/slide sheet, glide board, transfer belt, support sock). If a device was not available, it did not contribute towards the score developed.

### Statistical analysis

This exploratory analysis had two main parts. The first part was the estimation of the proportion of variance in step-rate occurring at each of four hierarchical levels (shifts within workers within wards within nursing homes). This was conducted using variance components analysis (VCA). VCA is a particular form of mixed-effects modelling that includes only random effects. As such, we constructed a mixed-effects linear regression model that included only these factors as random intercepts.

The second part of the analysis was the identification of potential determinants of step-rate at each of these four levels. We did this in two steps. First, we conducted a univariate analysis for each potential determinant by adding each determinant individually as a fixed-effect to the random-intercept model described above, and resolving the model. To investigate whether the associations identified in the univariate analysis were independent of each other (i.e. whether or not determinants share causal pathways) we then combined all significant (*P* < 0.05) determinants identified in the univariate procedure into a single multivariate model. We present *β*-coefficients with confidence intervals (CIs) and marginal *r*^2^ values for all models investigated. The marginal *r*^2^ value is an indicator of explained variance for the model’s fixed effects only (as opposed to both fixed and random effects together). We chose to provide the marginal *r*^2^ value as we were primarily interested in the explanatory power of the fixed-effect determinants in the model, rather than the model as a whole.

All statistical analyses were conducted in R (R Core Team, 2018)/RStudio (RStudio Team, 2016) using packages: lme4 (Bates *et al.*, 2019); VCA (Andre and Dufey, 2020); broom.mixed (Bolker *et al.*, 2020); DHARMa (Hartig, 2020); insight (Lüdecke *et al.*, 2020); and the tidyverse suite of packages (Wickham *et al.*, 2019).

## Results

For the organizational level variables, 17 (of 20) nursing home managers and all 42 ward managers (looking after 126 wards) completed the questionnaire. Of those not fully completing the questionnaire, two nursing home managers answered partly and one nursing home manager did not respond at all. Of the 553 participants (workers) included in DOSES, 452 (82%) provided data from the accelerometer measurements. Thirty participants were removed from the analysis due to not having a valid shift and a further two were participants removed due to not having a day or evening shift. This left 420 participants that contributed to this analysis with, in total, activity data for 1287 shifts. Participants were generally middle aged (mean [SD] = 46 [10.6]), women (95%) and rated their health as ‘good’ or better (85%) (Brazier *et al.*, 1992). Just under half (48%) were care aides, while most of the rest (42%) were care helpers. Most (75%) of the participants worked in somatic wards. The average (SD) length of the shifts was 7.1 (1.3) h and most were day shifts (68%). The average (SD) step-rate (grand mean across all shifts) was 1124 (315) steps per hour. Full details are presented in Table 1.

**Table 1.** Worker demographics and shift characteristics in elderly care.

Demographics	Mean (SD)	*n* (%)
Worker demographics (*n* = 420)		
Age (years)	46.0 (10.6)	—
Sex (female)	—	400 (95.2)
BMI (kg m^−2^) (*n* = 415)	26.4 (5.3)	—
Health (*n* = 414)		
Excellent	—	11 (2.7)
Very good	—	116 (28.0)
Good	—	224 (54.1)
Not so good	—	57 (13.7)
Poor	—	6 (1.4)
Job (*n* = 417)		
Care aide	—	200 (48.0)
Care helper	—	176 (42.2)
Nurse or other health professional	—	41 (9.8)
Type of ward		
Somatic	—	316 (75.2)
Dementia	—	82 (19.5)
Temporary rehabilitation	—	14 (3.3)
Independent living	—	8 (1.9)
Shift characteristics (*n* = 1287)		
Duration of shift (h)	7.1 (1.3)	—
Day (versus evening)	—	878 (68.2)
Weekday (versus weekend)	—	1010 (78.5)
Steps per hour at work	1124 (315)	—

BMI, body mass index.

### Proportion of variance in step-rate occurring at each level

In our sample of workers, variance in step-rate occurred primarily between shifts (i.e. day-to-day variability) and between workers. The proportion of total variance occurring from these two sources was 44.9 and 49.1%, respectively. This left only 6.1% of the variance to occur at the ward and nursing home levels, which contributed 0.5 and 5.6%, respectively.

### Potential determinants of step-rate at each level

In the univariate models (Table 2), significant determinants of step-rate occurred at all hierarchical levels (between shifts within workers, between workers within wards, between wards within nursing homes, and between nursing homes). At the shift level, weekend (versus weekday) and evening (versus day) shifts were associated with significantly higher step-rates (*β* = 89.7 [95% CI: 57.5; 121.8] and 129.6 [85.7; 173.8], respectively). This means that, for example, the model predicts that workers on an evening shift will have a step-rate 129.6 steps per hour higher than workers on a day shift. At an average shift duration of just over 7 h (7.1) this equates to roughly 920 more steps over the course of that shift.

**Table 2.** Shift, worker, ward, and nursing home level determinants of steps per hour at work.

Covariates	$r_{m}^{2}$	Univariate		Multivariate	
		*β* [95% CI]	*P* value	*β* [95% CI]	*P* value
Shift					
Duration of shift (h)	<0.01	−3.4 [−14.4; 7.7]	0.549		
Evening (versus day)	0.04	**129.6 [85.7; 173.8]**	**<0.001**	**99.7 [13.9; 186.5]**	**0.022**
Weekend (versus weekday)	0.01	**89.7 [57.5; 121.8]**	**<0.001**	**88.0 [55.7; 120.2]**	**<0.001**
Worker					
Age (years)	<0.01	0.9 [−1.5; 3.3]	0.474		
Sex (female)	<0.01	−106.3 [−225.9; 13.5]	0.082		
BMI (kg m^−2^)	<0.01	−0.6 [−5.4; 4.3]	0.822		
Country of birth (outside Denmark)	<0.01	47.7 [−19.0; 114.3]	0.159		
Steps per hour at leisure	0.02	**0.08 [0.03; 0.13]**	**<0.001**	**0.06 [0.01; 0.11]**	**0.018**
Job	0.02				
Care aide		ref	ref	ref	ref
Care helper		**103.4 [50.2; 156.7]**	**<0.001**	**111.4 [57.4; 165.5]**	**<0.001**
Nurse or other health professional		49.3 [−38.0; 136.7]	0.267	41.4 [−46.7; 129.6]	0.356
Time spent conducting					
Direct care tasks	0.01	**101.2 [25.8; 177.0]**	**0.008**	57.9 [−17.0; 132.9]	0.127
Support tasks	0.01	59.3 [−31.4; 150.0]	0.199		
Administration tasks	0.01	28.1 [−91.2; 147.8]	0.644		
Quantitative demands^a^	0.01	−1.1 [−2.4; 0.1]	0.076		
Influence at work^a^	<0.01	−0.7 [−2.0; 0.7]	0.323		
Social support^a^	<0.01	−0.4 [−1.9; 1.1]	0.617		
Quality of leadership^a^	<0.01	−0.7 [−2.3; 0.9]	0.402		
Ward					
Ward type	0.02				
Somatic		ref	ref	ref	ref
Dementia		**−113.0 [−180.2; −45.9]**	**0.001**	**−92.5 [−166.3; −18.3]**	**0.013**
Temporary rehabilitation		−21.3 [−173.7; 131.2]	0.783	−42.0 [−207.4; 124.2]	0.614
Independent living		−61.9 [−261.1; 137.6]	0.542	−47.0 [−236.8; 146.5]	0.626
Ward size (max number of residents)	0.01	5.6 [−1.4; 12.6]	0.117		
Resident–staff ratio (usual number of residents/usual number of staff)	0.03	**27.3 [16.8; 38.0]**	**<0.001**	1.7 [−19.1; 22.4]	0.868
Rooms to take breaks	<0.01	5.6 [−98.9; 111.3]	0.915		
Allowed to take breaks	0.02	**132.4 [53.4; 212.5]**	**0.001**	71.6 [−13.4; 159.5]	0.100
Location of aides^b^	<0.01	−34.0 [−167.0; 105.6]	0.617		
Nursing home					
Size (max *n* residents)	<0.01	−0.3 [−1.8; 1.3]	0.697		
Number of floors	0.01	39.1 [−1.7; 78.8]	0.059		
Elevators (yes)	0.01	**−133.4 [−245.4; −15.6]**	**0.025**	−118.4 [−236.1; 11.0]	0.057

$r_{m}^{2}=marginal\;r^{2}$ ; the fixed effects variance divided by the total variance. The multivariate $r_{m}^{2}=0.12$. Double = shift spanned both day and evening. BMI, body mass index.

^*a*^Measured as a 0–100 point scale.

^*b*^Continuous variable created as an average from Likert scales denoting the stored location of aides that were available for use: 4-point scale from ‘in the room’ to ‘in a different ward’.

At the worker level, a higher step-rate at work was associated with a higher step-rate at leisure (*β* = 0.08 [0.03; 0.13]), with being a care helper (compared with a care aide; *β* = 103.4 [50.2; 156.7]) and with having a larger proportion of work involving direct care tasks (*β* = 101.2 [25.8; 177.0]). At the ward level, working in a dementia ward, compared with a somatic ward, was associated with a lower step-rate at work (*β* = −113.0 [−180.2; −45.9]). On the other hand, an increased resident–staff ratio and being allowed to take breaks during your shift were associated with an increase in step-rate at work (*β* = 27.3 [16.8; 38.0] and 132.4 [53.4; 212.5], respectively). Finally, at the nursing home level, the presence of elevators was associated with a decrease in step-rate at work (*β* = −133.4 [−245.4; −15.6]).

The marginal *r*^2^ values (i.e. *r*^2^ values for the fixed-effects only) for each potential determinant ranged from <0.01 to 0.04 with the highest values found for the type of shift (day/evening) and the resident–staff ratio. Full details for all potential determinants tested are presented in Table 2.

When combined in the multivariate analysis, several potential determinants markedly changed their effect estimates (Table 2). This suggests they share a causal chain with other factors assessed in the model. These were, the proportion of time spent conducting direct care tasks (change (Δ) *β* = 43.3), the resident–staff ratio (Δ*β* = 25.6), being allowed to take breaks (Δ*β* = 60.8). Determinants that maintained a significant association with step-rate at work in the multivariate model were weekend shifts (*β* = 88.0 [55.7; 120.2]), evening shifts (compared with day shifts; *β* = 99.7 [13.9; 186.5]), step-rate during leisure (*β* = 0.06 [0.01; 0.11]), and being a care helper (*β* = 111.4 [57.4; 165.5]). Working in a dementia ward (compared with a somatic ward) was associated with a lower step-rate (*β* = −92.5 [−166.3; −18.3]). The marginal *r*^2^ value for the complete multivariate model was 0.12.

## Discussion

### Summary of findings

This multi-level study identified that eldercare workers took, on average (SD), 1124 (315) steps per hour and that nearly all the variability in step-rate at work occurred between shifts (i.e. day-to-day variability within workers) and between workers, encompassing ~45 and ~49% of the total variance, respectively. We found that several determinants were significantly associated with a higher step-rate. At the shift level, these were weekend and evening shifts (versus weekday/day). At the worker level, these were having a job as a care helper (versus care aide), an increased proportion of time spent on direct care tasks and a higher step-rate during leisure. At the ward level, these were working in a somatic ward (versus dementia), an increased resident–staff ratio and permission to take unscheduled breaks. Finally, at the nursing home level, the presence of elevators was associated with a lower step-rate at work.

### Strengths and limitations

The major strengths of this study are the large sample size, the use of accelerometers for measuring step-rate over multiple days, and the multi-level design that allowed us to take into account effects at the hierarchical levels assessed (shift, worker, ward, and nursing home). The primary limitation of this study is its cross-sectional nature, which limits our ability to make causal inferences from the results obtained. The choice to take a more exploratory approach to this analysis was because of the relative sparsity of literature on this topic. Other limitations are the use of a convenience sample of workers, wards, and homes, and that we do not have information about the temporal distribution of steps within a shift, which may be of importance for the effects of step-rate on fatigue and health (Mathiassen, 2006).

### Comparisons with other studies

Although no previous studies have investigated the variety of organizational factors in eldercare workers that we do, a few studies have assessed particular organizational determinants of OPA, which allows us to compare findings. These studies seem to be in agreement with our own for some of the investigated factors. For instance, the social environment (e.g. social support) does not seem to be related to OPA (Sawyer *et al.*, 2017; Clays *et al.*, 2020) and management discouragement of breaks seems to be associated with a decreased OPA (Sawyer *et al.*, 2017). On the other hand, Takahashi *et al.* found that longer working shifts were associated with lower intensities of OPA (Takahashi *et al.*, 1999). However, in that study the shifts associated with lower intensities of OPA (i.e. the longer shifts) were all night shifts—a shift-level characteristic that was not investigated in our study. Of note, though, the studies cited above (Sawyer *et al.*, 2017; Clays *et al.*, 2020), investigated different populations to our own. These differences mean that comparing results between these studies and our own requires considerations as to how similar the populations are and whether the seemingly similar factors do, indeed, represent similar effects. For example, Sawyer *et al.* (2017) conducted their study in office workers and the activity conducted during a ‘break’ seems likely to be quite different between office workers and eldercare workers.

### Meaning and implications

The determinants that had the greatest explanatory strength (as measured through the $r_{m}^{2}$ value) were organizational determinants (i.e. day versus evening shifts and the resident–staff ratio) as opposed to individual determinants. Although we did identify one individual determinant that was related to step-rate at work (step-rate at leisure; indicating that eldercare workers who take more steps during leisure also take more steps at work), this result is less useful when considering how organizations can modify the step-rate of their workers to improve health. That said, the positive association between steps at work and during leisure may be a sign that some workers are generally more fit, thus having energy to walk more both at work and after work, and that working conditions may influence both fitness and energy.

The organizational determinants that we identified as related to step-rate at work were the type of shift (evening/weekend shift), the type of tasks required (job as a care helper, proportion of time spent on direct care tasks, working in a dementia ward) and factors related to the workload (the resident–staff ratio, being allowed to take breaks, the presence of elevators). As such, our results suggest that modifying these factors is likely to have an effect on step-rate at work. The finding of an association between being permitted to take breaks at work and having more steps is particularly interesting, as the opposite result may be more intuitive. However, a possible explanation of the ‘inverted’ association could be that workers in busy wards, where caretaking requires extensive walking, are, to a larger extent than workers in less busy wards, permitted to take rest breaks at their discretion, as needed for recovery. Our results may be used to help prevent fatigue, pain, and sick leave by reducing steps among those taking too many steps, and to promote health by increasing steps among those workers sitting too much. For example, increasing the number of staff per shift would decrease the resident–staff ratio, which, in turn, is likely to decrease the step-rate of workers. Unfortunately, we cannot say what might be too many, or too few, steps as such speculation is beyond the scope of this study. However, this information is vital when considering the development and implementation of interventions to modify step-rate.

This study showed that the differences in step-rate at work occurred primarily between shifts (within workers) and between workers (within wards), with only a small amount occurring between wards (within nursing homes) and between nursing homes. The smaller variance in steps explained by higher levels could be due to sampling of nursing homes with similar organizational characteristics. It is also possible that effects of higher level determinants are acting on step-rate through lower levels. This could be due to unidentified modifying factors. For example, the presence or absence of elevators in nursing homes will generally affect the step-rate of all workers within that nursing home. However, it seems likely that whether or not a ward spans multiple floors (i.e. whether or not the workers in that ward need to change floors) will modify the effect of elevators on the step-rate of workers—creating differences in step-rate that occurs between wards. Such interactions need to be addressed in future research.

### Future research

The exploratory nature of the present research means that replication of our findings is required. Furthermore, some of the determinants identified are not modifiable (e.g. weekend/evening shifts). In those cases, we need a greater understanding of what it is about weekend/evening shifts that influences step-rate. For example, the resident–staff ratio is higher on evening shifts. The resident–staff ratio is a modifiable factor that may, thus, in part, account for difference in step-rate between, e.g. day and evening shifts. Finally, the variance in step-rate explained by the investigated factors (in terms of marginal *r*^2^ values) was low, suggesting that other (unmeasured) determinants make an important contribution to step-rate in this population.

## Conclusion

This study identified an average (SD) occupational step-rate among eldercare workers of 1124 (315) steps per hour, and that nearly all of the variance in step-rate in eldercare work occurred between shifts (~45%) and between workers (~49%). A higher step-rate at work was associated with the type of shift (weekend/evening shift versus weekday/day), the type of tasks required (job as a care helper versus care aide, an increased proportion of time spent on direct care tasks and working in a somatic ward (versus dementia) and the workload (an increased resident–staff ratio, being allowed to take breaks and the lack of elevators). These areas provide key targets within eldercare work that may be used to modify the step-rate for workers in this sector.
